# Pharmacological degradation of ATR induces antiproliferative DNA replication stress in leukemic cells

**DOI:** 10.1002/1878-0261.13638

**Published:** 2024-03-22

**Authors:** Anita G. Kansy, Ramy Ashry, Al‐Hassan M. Mustafa, Abdallah M. Alfayomy, Markus P. Radsak, Yanira Zeyn, Matthias Bros, Wolfgang Sippl, Oliver H. Krämer

**Affiliations:** ^1^ Institute of Toxicology University Medical Center of the Johannes Gutenberg University Mainz Mainz Germany; ^2^ Department of Oral Pathology, Faculty of Dentistry Mansoura University Egypt; ^3^ Department of Zoology, Faculty of Science Aswan University Egypt; ^4^ Department of Medicinal Chemistry, Institute of Pharmacy Martin‐Luther‐University of Halle‐Wittenberg Halle (Saale) Germany; ^5^ Department of Pharmaceutical Chemistry, Faculty of Pharmacy Al‐Azhar University Assiut Egypt; ^6^ 3^rd^ Department Medicine University Medical Center Mainz Germany; ^7^ Department of Dermatology University Medical Center Mainz Germany; ^8^ Present address: Medical Deptpartment VII Donau‐Isar‐Klinikum Deggendorf Germany.

**Keywords:** ATR, cereblon, DNA replication stress, leukemia, PROTAC

## Abstract

Mammalian cells replicate ~ 3 × 10^9^ base pairs per cell cycle. One of the key molecules that slows down the cell cycle and prevents excessive DNA damage upon DNA replication stress is the checkpoint kinase ataxia‐telangiectasia‐and‐RAD3‐related (ATR). Proteolysis‐targeting‐chimeras (PROTACs) are an innovative pharmacological invention to molecularly dissect, biologically understand, and therapeutically assess catalytic and non‐catalytic functions of enzymes. This work defines the first‐in‐class ATR PROTAC, Abd110/Ramotac‐1. It is derived from the ATR inhibitor VE‐821 and recruits the E3 ubiquitin‐ligase component cereblon to ATR. Abd110 eliminates ATR rapidly in human leukemic cells. This mechanism provokes DNA replication catastrophe and augments anti‐leukemic effects of the clinically used ribonucleotide reductase‐2 inhibitor hydroxyurea. Moreover, Abd110 is more effective than VE‐821 against human primary leukemic cells but spares normal primary immune cells. CRISPR‐Cas9 screens show that ATR is a dependency factor in 116 myeloid and lymphoid leukemia cells. Treatment of wild‐type but not of cereblon knockout cells with Abd110 stalls their proliferation which verifies that ATR elimination is the primary mechanism of Abd110. Altogether, our findings demonstrate specific anti‐leukemic effects of an ATR PROTAC.

## Graphical abstract

Hydroxyurea depletes the cellular dNTP pools and causes DNA replication stress. The checkpoint kinase ATR protects cells from DNA replication catastrophe and apoptosis upon drug‐induced DNA replication stress. In leukemic cells from various origins, the PROTAC Abd110/Ramotac‐1, which is based on the ATR inhibitor VE‐821 and the E3 ubiquitin‐ligase component CRBN, exerts synergistically lethal effects with hydroxyurea.

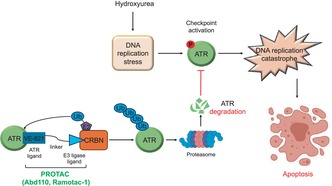

**Abbreviations**
Abd110: ATR PROTAC Ramotac‐1
AML: acute myeloid leukemia
ATM: ataxia telangiectasia‐mutated
ATR: ataxia‐telangiectasia‐and‐RAD3‐related
B‐/T‐ALL: B‐/T‐cell acute lymphocytic leukemia
CD: cluster of differentiation
CHK1: checkpoint kinase‐1
CHK2: checkpoint kinase‐2
cl.: cleaved
CRBN: cereblon
DNA‐PKcs: DNA‐dependent protein kinase catalytic subunit
DSBs: double‐stranded DNA breaks
ɣH2AX: H2AX phosphorylated at S139
kDa: kilo Dalton
MPN: myeloproliferative neoplasm
p‐: phosphorylated
PBMCs: peripheral blood mononuclear cells
PROTAC: proteolysis‐targeting‐chimera
sgRNA: small guide RNA

## 1 Introduction

Chemotherapeutics kill tumor cells through the induction of DNA replication stress and DNA damage. The checkpoint kinases ataxia telangiectasia‐mutated (ATM), checkpoint kinase‐2 (CHK2), and DNA‐dependent protein kinase catalytic subunit (DNA‐PKcs) are activated in cells with double‐stranded DNA breaks (DSBs). Stalled DNA replication forks and single‐stranded DNA breaks primarily activate the checkpoint kinases ATM‐and‐RAD3‐related (ATR) and checkpoint kinase‐1 (CHK1). Induced checkpoint kinases orchestrate cell cycle progression, DNA replication origin firing, DNA repair ensuring genome integrity, and cell fate [1, 2, 3].

Cancer cells often have high endogenous levels of DNA replication stress and consequently rely on checkpoint kinases [1, 2, 3]. This notion has propelled an intense search for their pharmacological inhibitors. Preclinical and clinical studies demonstrate that ATP‐competitive ATR inhibitors kill subsets of tumor cells [4, 5]. Proteolysis‐targeting‐chimeras (PROTACs) are novel, highly promising heterobifunctional molecules [2, 6]. They contain a ligand for the protein of interest, a ligand binding an E3 ubiquitin‐ligase, and a linker connecting these ligands. PROTACs direct the ubiquitination machinery to their targets. Consequently, these become polyubiquitinated and degraded by proteasomes. Advantages of PROTACs over corresponding small molecule inhibitors include depletion of their targets and often enhanced potency and selectivity. Eighteen protein degraders are in phase I‐III clinical trials for cancer treatment [7]. Here we characterize Abd110/Ramotac‐1 as first‐in‐class PROTAC for ATR.

## 2 Materials and methods

### 2.1 PROTACs and control compounds

All new agents and their underlying building blocks were synthesized by the group of Prof. Wolfgang Sippl, Halle, Germany.

### 2.2 Cell culture and primary cells

Cells were cultured at 37 °C and 5% CO_2_ in RPMI (Thermo Fisher, Gibco, Braunschweig, Germany) supplemented with 5% FCS (Sigma‐Aldrich, Munich, Germany) and 1% Penicillin/Streptomycin (Thermo Fisher). Primary cells were cultivated in IMDM (Thermo Fisher) supplemented with 20% FCS, 1% penicillin/streptomycin, 1xMEM non‐essential amino acids (Euroclone, Pero, Italy) and 10 ng·mL^−1^ Interleukin‐3. Prof. Dr. F.‐D. Böhmer and Prof. Dr. T. Heinzel, Jena, Germany gave us leukemic cells (originally from the DSMZ, authenticated by DNA fingerprint profiling using eight different and highly polymorphic short tandems repeats, at the Leibniz‐Institute DSMZ, Braunschweig, Germany). These were MOLT_4 (RRID:CVCL_0064), RS4‐11 (CVCL_0093), MV4‐11 (RRID:CVCL_0064), MOLM‐13 (RRID:CVCL_2119), HL‐60 (RRID:CVCL_0002), and SET‐2 (RRID:CVCL_2187). Prof. Dr. G. Winter, Vienna, Austria gave us verified MOLT‐4 cells wild‐type and cereblon (CRBN) null cells. MOLT‐4 CRBN−/− were generated by CRISPR‐Cas9 [8]. All cell lines were regularly tested negative for mycoplasma using an enzymatic assay kit. The Blood transfusion unit of the University Medical Center Mainz provided us buffy coats for the isolation of peripheral blood mononuclear cells (PBMCs). PBMCs were isolated from buffy coats of four healthy donors (tested negative for common infections) using Biocoll (Bio&Sell, Feucht, Nuremburg, Germany). Control cells and treated cells were incubated with antibodies to discriminate lineage markers by flow cytometry. The cells were defined with antibodies for lineage‐specific cell surface markers: CD3^−^CD19^+^ as B cells; CD3^+^ as T cells, CD3^−^CD19^−^CD14^+^ as monocytes; CD3^−^CD19^−^CD1c^+^ as dendritic cells; CD3^−^CD19^−^CD56^+^ as natural killer (NK) cells; and CD3^−^CD14^−^CD19^−^CD56^−^CD11b^+^ as PMNs. Cell viability was evaluated using annexin‐V AF647 (#A23204; early apoptosis marker) and FVD eFl780 (#65‐0865‐18; late apoptosis marker; both from ThermoFisher). The following antibodies were used: CD11b BV510 (#101263), CD1c BV605 (#331538), CD3 BV711 (#344838) from BioLegend, San Diego, CA, USA; CD14 PE‐eFl610 (#61‐0149‐42), CD56 Pe‐Cy7 (#25‐0567‐42), CD19 AF488 (#53‐0199‐42) from ThermoFisher. The graphs were created with graphpad prism 6.0 (Graphpad software inc., La Jolla, CA, USA). Experiments with patient samples have ethic approval by the Landesärztekammer Rhineland‐Palatine no.: 837.258.17 (11092).

### 2.3 Flow cytometry

Flow cytometry was performed as recently noted by us [9, 10, 11]. Annexin‐V (indicator of early apoptosis; #130‐093‐060) was from Miltenyi Biotec, Bergisch Gladbach, Germany, and propidium iodide (PI; late apoptosis indicator) was from Sigma‐Aldrich.

### 2.4 Immunoblotting and immunofluorescence

Details on immunoblotting were summarized by us [9, 10, 11]. The following antibodies were used: ATR (2790S), CHK1 (2360), cleaved caspase‐3 (9661), CRBN (71810), PARP1 (9542) and p‐CHK1_(S345)_ from Cell Signaling, Leiden, Netherlands; ATM (ab32420), DNA‐PKcs (ab32566) and p‐ATM_(T1981)_ (ab81292) from Abcam, Cambridge, UK; GSPT1 (sc‐515 615), HSP90 (sc‐13 119) and vinculin (sc‐73 614) from Santa Cruz, Heidelberg, Germany; p‐ATR_(T1989)_ (GTX128145) from GeneTex, Irvine, MA, USA; ɣH2AX_(S139)_ (MAB3802) from Millipore, Schwalbach, Germany. The protein ladders were the prestained Scientific™ PageRuler™ (#26616) and the prestained Scientific™ PageRuler™ Plus (#26619) from Thermo Fisher.

### 2.5 DepMap database analysis

The DepMap project (collaborative effort of Broad Institute and Wellcome Sanger Institute) has analyzed large‐scale datasets to define a landscape of genetic targets for therapeutic development. ATR was eliminated by CRISPR‐Cas9, and 14 days later pooled cells were examined for sgRNA expression. 0 means no impact, negative values indicate dependency (https://forum.depmap.org/t/depmap‐genetic‐dependencies‐faq/131).

## 3 Results and Discussion

### 3.1 Abd110 induces proteasomal degradation of ATR through CRBN

**Fig. 1:**
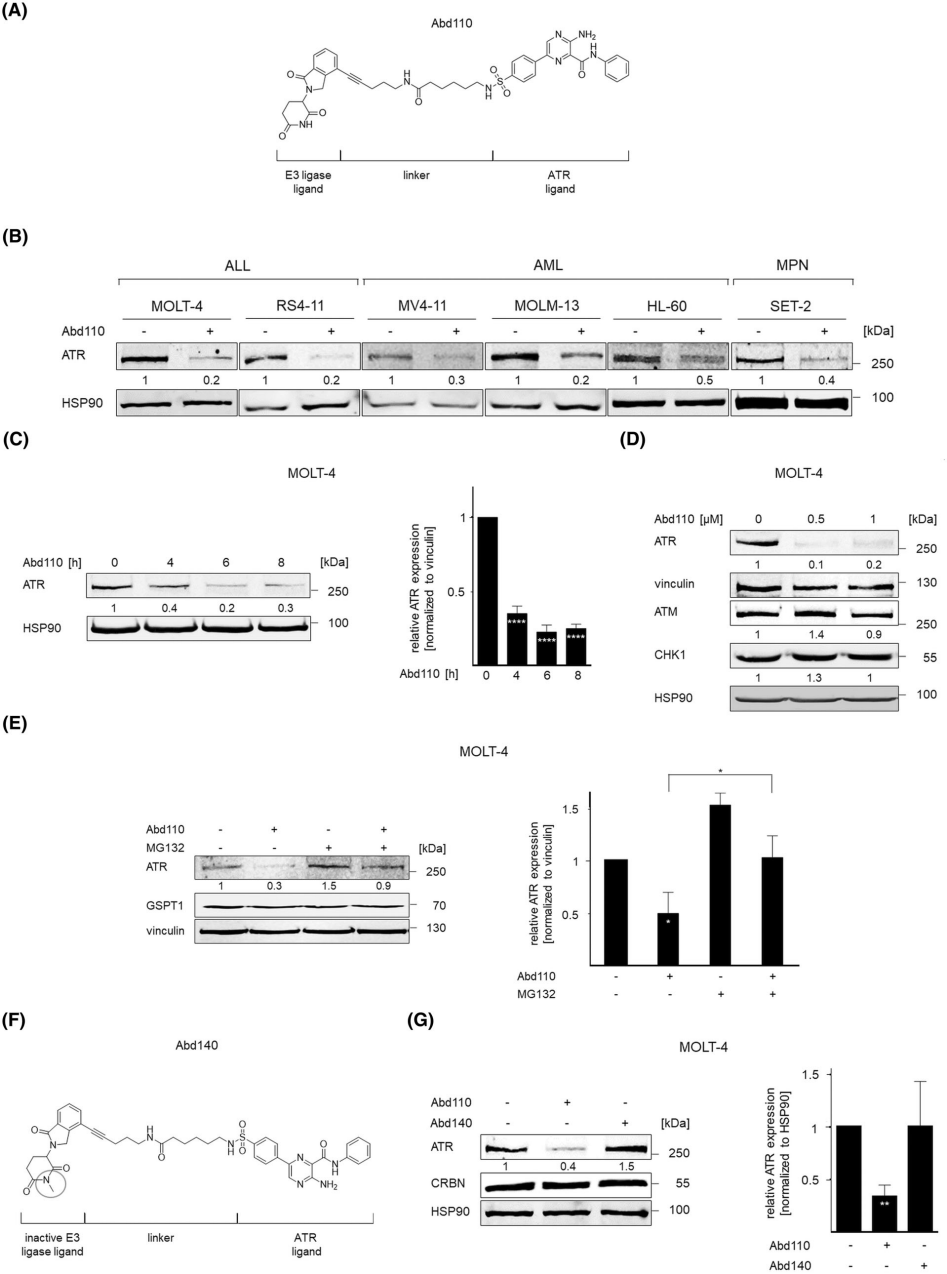
Abd110 depletes ATR in leukemic cells. (A) Chemical structure of Abd 110, composed of lenalidomide (left, CRBN ligand), a linker, and the ATR‐binding ligand (VE‐821, right). (B) The indicated cell types were treated with 1 μm Abd110 for 24 h. Immunoblots were carried out for the indicated proteins; HSP90 as loading control (*n* = 2). The Odyssey system was used to quantify the band intensities, ATR signals were normalized to the loading control, untreated cells set as 1 in all figures. (C) Cells were treated with 1 μm Abd110 for 0, 4, 6, and 8 h. Immunoblot (left) was carried out with HSP90 as loading control. Bar chart (right) shows ATR levels after treatment, ****P < 0.0001; Bonferroni's multiple comparison test; created with graphpad prism 6.0 (*n* = 4). (D) Cells were treated with 0.5–1 μm Abd110 for 24 h. Immunoblot was done for vinculin and HSP90 as loading controls (*n* = 2). (E) Cells were incubated with 0.5 μm Abd110 and 0.5 μm MG132 for 4 h. Immunoblot (left) was carried out as indicated, vinculin and HSP90 are loading controls. Bar chart (right) shows ATR levels; *P < 0.1, Bonferroni's multiple comparison test; created with graphpad prism 6.0 (*n* = 3). (F) Chemical structure of Abd140 which corresponds to Abd110; the methyl group (green circle) prevents binding to CRBN. (G) Cells were treated with 1 μm Abd110 or Abd140 for 24 h. Immunoblots (left) were carried out as indicated; HSP90 as loading control. Bar chart (right) shows ATR levels; **P < 0.01; Bonferroni's multiple comparison test; created with graphpad prism 6.0 (*n* = 4).

We set out to identify a degrader of ATR, which is a protein with a long half‐life of about 24 h [1, 2, 3]. We have recently described the chemical synthesis, *in vitro* activity, and stability of Abd110 (named 42i in [12]) which is based on the ATR inhibitor VE‐821 and lenalidomide. Lenalidomide recruits the E3 ubiquitin‐ligase component CRBN to its binding partners (Fig. 1A). To characterize the biological effects of Abd110 across various cellular systems, we incubated cells from acute lymphocytic leukemia (ALL), acute myeloid leukemia (AML), and essential thrombocytopenia (myeloproliferative neoplasm, MPN) patients with 1 μm Abd110 for 24 h. Immunoblot analyses revealed that Abd110 reduced ATR by 70–90% (Fig. 1B).

Time course analyses showed that a 4‐h incubation with 1 μm Abd110 sufficed to reduce ATR significantly to 30% of its levels in untreated cells (Fig. 1C). A concentration of 0.5 μm Abd110 eliminated 90% of ATR after 24 h (Fig. 1D).

To exclude effects of Abd110 on ATR‐related kinases, we probed immunoblots for ATM and CHK1. Abd110 decreased ATR without altering the structurally related ATM and CHK1 (Fig. 1D). PROTACs can target physiologically relevant neosubstrates but Abd110 contains a modified lenalidomide scaffold that should reduce such off‐target effects [13]. Indeed, Abd110 did not decrease the pro‐proliferative CRBN neosubstrate G1‐to‐S‐phase‐transition‐protein‐1 (GSPT1/eRF3a/b) (Fig. 1E). These data illustrate specificity of Abd110.

The pharmacology of PROTACs relies on induced proteasomal degradation [7]. To examine if Abd110 triggers proteasomal degradation of ATR, we used MG132. This well‐established proteasome inhibitor halted the Abd110‐induced loss of ATR significantly (Fig. 1E), verifying the proteasomal degradation mechanism.

To examine the functional role of CRBN for the Abd110‐induced ATR degradation, we designed Abd140 (named 42 m in [12]). This Abd110 analogue cannot bind CRBN due to a methylated glutarimide (Fig. 1F) and hence represents a suitable negative control. Abd140 did not attenuate ATR (Fig. 1G), which confirms that Abd110 is a CRBN‐dependent ATR degrader.

### 3.2 Abd110 augments DNA replication stress and DNA damage

**Fig. 2:**
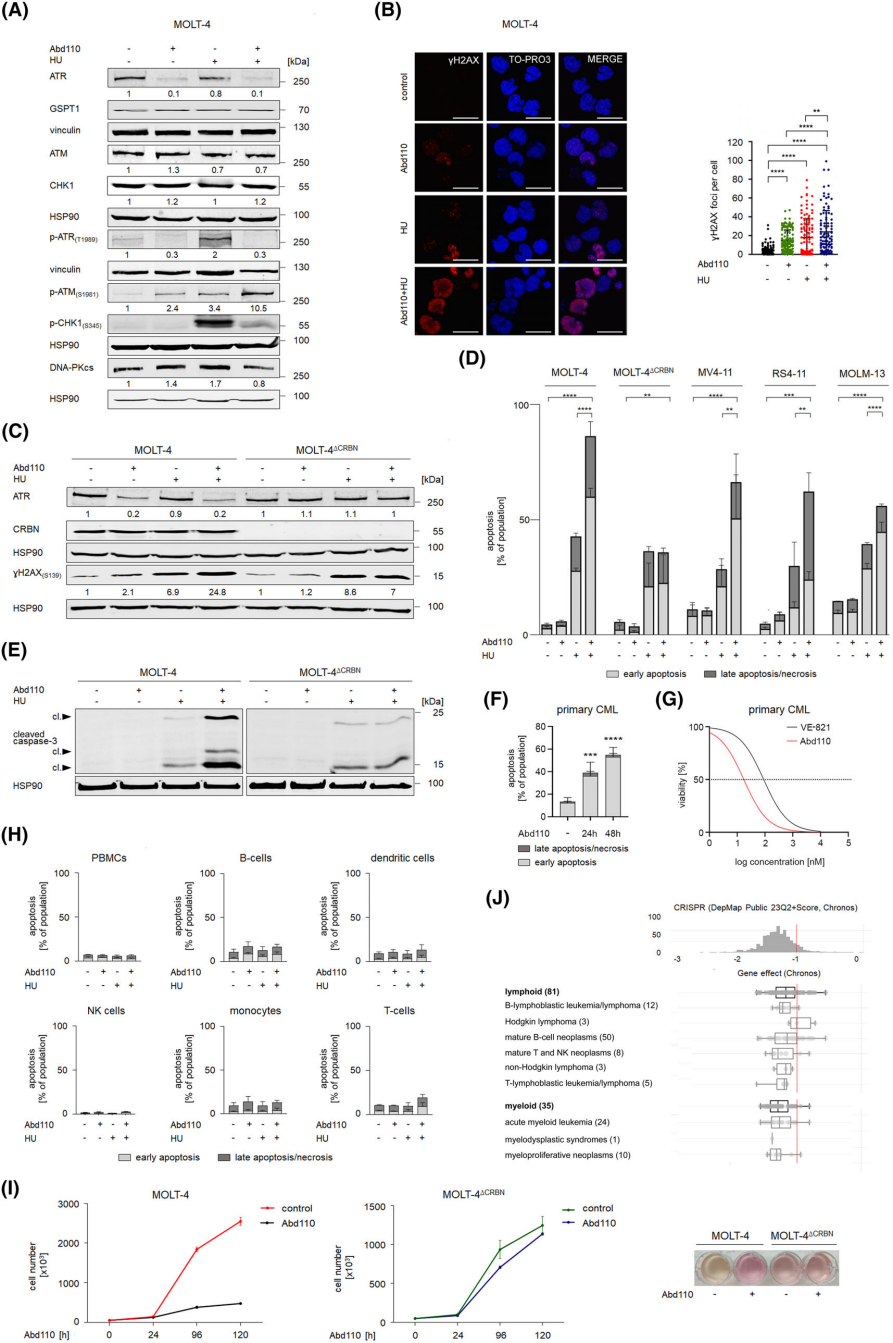
Abd110 enhances DNA injury and cell death upon hydroxyurea‐induced DNA replication stress. (A) Cells were treated with 0.5 μm Abd110 for 24 h, 1 mm hydroxyurea (HU) was added for the last 4 h. Immunoblots were done as indicated; vinculin and HSP90 as loading controls (*n* = 3). The Odyssey system was used to quantify band intensities, ATR signals were normalized to the loading control, untreated cells set as 1 in all figs. (B) Cells were treated as mentioned in A. Immunofluorescence staining (left) was carried for S139‐phosphorylated H2AX (ɣH2AX_(S139)_), TO‐PRO‐3 for nuclear staining; scale bar 20 μm. Bar chart (right) shows quantification of ɣH2AX foci as dot blot; **P < 0.01, ****P < 0.0001; Bonferroni's multiple comparison test; created with graphpad prism 6.0 (*n* = 3). (C) MOLT‐4 and MOLT‐4^ΔCRBN^ cells were treated with 0.5 μm Abd110 for 24 h and 1 mm HU for 4 h. Immunoblots were done as indicated with HSP90 as loading control (*n* = 3). (D) The indicated cells were treated with 1 μm Abd110 and 1 mm HU for 24 h. Cells were stained with annexin‐V/PI; **P < 0.01; ***P < 0.001; ****P < 0.0001, Bonferroni's multiple comparison test, early plus late apoptosis; created with graphpad prism 6.0 (*n* = 3). (E) Cells were incubated as mentioned in D. Immunoblots show caspase‐3 and HSP90 as loading control. The bands revealed with the cleaved caspase‐3 antibody are active caspase‐3 lacking its autoinhibitory domain (cl., cleaved; *n* = 2). (F) Primaries were treated with 0.5 μm Abd110 for 24–48 h. Cells were stained with annexin‐V/PI; ***P < 0.001; ****P < 0.0001, Bonferroni's multiple comparison test, early plus late apoptosis; created with graphpad prism (*n* = 3). (G) Primaries were treated with 1, 10, 100, 1000, and 10 000 nm Abd110 or VE‐821 for 24 h and stained with annexin‐V/PI. Cell viability was plotted against logarithmic drug concentrations to determine IC_50_ values, created with graphpad prism 6.0 (*n* = 2). (H) PBMCs were treated with 0.5 μm Abd110 and 1 mm HU for 24 h and subjected to flow cytometry assessing apoptosis (*n* = 4; mean + SD). Control cells and Abd110 ± hydroxyurea‐treated cells were incubated with antibodies specific for lineage markers and subjected to flow cytometry (details on antibodies are provided in the supplementary file). (I) Growth of MOLT‐4 and MOLT‐4^∆CRBN^ cells after treatment with 0.5 μm Abd110 for 24–120 h; 10^5^ cells·mL^−1^ were seeded at 0 h; created with graphpad prism 6.0 (*n* = 3; mean ± SD). Image of the cells was taken after treatment with 0.5 μm Abd110 for 144 h (right). (J) Data retrieved from the DepMap project illustrate that 116 myeloid and lymphoid cancer cell lines rely on ATR. The dependency cutoff is at −1 (red line). Dependency scores below 0 indicate that cells rely on the analyzed factor. The more negative a score is for a gene, the more essential it is in a given cell line. Common essential genes have dependency scores of −1 and less (i.e., 100% counterselection against the cells with the knockdown, growth arrest, or killing; https://forum.depmap.org/t/depmap‐genetic‐dependencies‐faq/131).

Next, we investigated if Abd110 augmented chemotherapy‐induced DNA damage. Hydroxyurea is clinically used to reduce excessive blast counts in leukemic patients. This ribonucleotide reductase‐2 inhibitor depletes cellular dNTP pools, stalls DNA replication forks, and causes the accumulation of single‐stranded DNA that is protected by replication‐protein‐A [14]. Such structures activate ATR to prevent their breakdown into DSBs being the most cytotoxic DNA lesion [3, 4]. We asked if Abd110 triggered replication catastrophe (conversion of stalled DNA replication forks into DSBs) [14]. Phosphorylation at T1989 activates ATR [1, 2]. Abd110 eliminated ATR and hydroxyurea‐induced pT1989‐ATR up to 90% in MOLT‐4 T‐ALL cells. ATM, CHK1, and DNA‐PKcs were unaffected. Hydroxyurea induces an ATR‐catalyzed phosphorylation of CHK1 at S345 [9]. Consistent with degradation of ATR, Abd110 suppressed this phosphorylation of CHK1 (Fig. 2A). We further noted that Abd110 plus hydroxyurea induced pS1981‐ATM more strongly than the single treatments (Fig. 2A). This accumulation of pS1981‐ATM corresponds to the accumulation of DSBs upon replication catastrophe [2, 4].

To extend these investigations, we studied MOLT‐4 cells by immunofluorescence for ɣH2AX foci indicating sites of DNA damage [14]. We limited incubation times with hydroxyurea to 4 h to avoid false‐positive results due to cell death‐associated DNA fragmentation [15]. Abd110 and hydroxyurea induced ɣH2AX foci, and Abd110 plus hydroxyurea further increased ɣH2AX signal intensities significantly. This was associated with nuclear fragmentation (Fig. 2B). These data disclose that pharmacological elimination of ATR provokes replication catastrophe.

To substantiate that Abd110 causes replication catastrophe through eliminating ATR, we used MOLT‐4 and corresponding CRBN‐deficient MOLT‐4^ΔCRBN^ cells [8]. We treated them with hydroxyurea and Abd110. Immunoblot analyses demonstrated that Abd110 increased ɣH2AX levels only in CRBN‐proficient cells. Likewise, Abd110 augmented hydroxyurea‐induced accumulation of ɣH2AX in MOLT‐4 cells but not in MOLT‐4^ΔCRBN^ cells. Hydroxyurea and Abd110 did not alter the expression of CRBN. To obtain genetic evidence for the CRBN‐dependent degradation of ATR by Abd110, we again used MOLT‐4 and MOLT‐4^ΔCRBN^ cells. Abd110 decreased ATR in untreated and hydroxyurea‐treated MOLT‐4 but not in MOLT‐4^ΔCRBN^ cells (Fig. 2C). This finding verifies the on‐target activity of Abd110 and corroborates data shown in Fig. 1G.

### 3.3 Abd110 eliminates leukemic cells by apoptosis

Checkpoint kinase inhibition upon DNA replication stress can cause synergistic anti‐tumor effects [1, 2]. We incubated MOLT‐4, MOLT‐4^ΔCRBN^ T‐ALL, MV4‐11 AML, RS4‐11 B‐ALL, and MOLM‐13 AML cells with 1 μm Abd110 plus 1 mm hydroxyurea; such doses of hydroxyurea are achievable in patients [6]. We tested for apoptosis (programmed cell death) using flow cytometry. Abd110 did not compromise cell vitality after 24 h (Fig. 2D). Thus, the reduction of ATR by Abd110 is not a consequence of cell killing. Hydroxyurea induced 28–43% apoptosis. Abd110 plus hydroxyurea synergistically augmented hydroxyurea‐induced apoptosis from 61 to 87% in MV4‐11, RS4‐11, MOLM‐13, and MOLT‐4 cells (Fig. 2D). Although hydroxyurea induced up to 36% apoptosis in MOLT‐4^∆CRBN^ cells, Abd110 did not augment this cytotoxic effect (Fig. 2D). Hence, Abd110 acts solely and specifically as CRBN‐dependent ATR degrader. Limited proteolysis of caspase‐3 generates the ultimate apoptosis executioner active caspase‐3 [6]. Coherent with Fig. 2D, immunoblotting confirmed that hydroxyurea activated caspase‐3 in MOLT‐4 and MOLT‐4^∆CRBN^ cells, and that Abd110 specifically increased this process in MOLT‐4 cells (Fig. 2E).

We asked if Abd110 is effective against primary cells from a 39‐year‐old female with first chronic phase *t(9;22)*‐positive chronic myeloid leukemia (CML). The patient was diagnosed with CML carrying the *BCR‐ABL* transcript *b2a2* and was treated with hydroxyurea for cytoreduction. Flow cytometry showed that Abd110 induced apoptosis in CD34^+^ patient cells *ex vivo* up to 55% after 24–48 h (Fig. 2F). Repeated addition of hydroxyurea was not necessary to achieve this effect. We compared the pro‐apoptotic efficacies of Abd110 versus its parent compound in these primaries. We found IC_50_ values of 17.3 ± 5.04 nm for Abd110 and 90.14 ± 4.11 nm for VE‐821, suggesting that the ATR PROTAC is 5.2‐fold more effective (Fig. 2G). These data illustrate that a sequential treatment with hydroxyurea and Abd110 eliminates primary leukemic cells.

To assess effects of Abd110 on primary normal immune cells, we used PBMCs. Hydroxyurea ± Abd110 did not kill such cells (Fig. 2H). We validated this observation in dendritic cells, B cells, monocytes, T cells, and natural killer cells that we separated with lineage‐specific antibodies (Fig. 2H). These data correspond to the finding that high levels of DNA replication stress occur in leukemic but not in normal cells [1, 2, 3].

The Abd110‐induced depletion of ATR and the subsequent accumulation of DNA lesions, indicated by ɣH2AX and pS1981‐ATM, did not kill leukemic cells after 24–48 h (Fig. 2B–E). Flow cytometry showed that Abd110 increased the numbers of cells in G1 phase (Fig. S1). Such slower cell cycle progression can prolong the time for DNA repair antagonizing cell death induction [2]. This can explain why Abd110 causes replication stress but not apoptosis. Nonetheless, Abd110 stalled the proliferation of MOLT‐4 but not MOLT‐4^ΔCRBN^ cells when given for prolonged times (Fig. 2I). This notion corresponds to data from the Cancer‐Dependency‐Map project that identify ATR as essential gene in 116 cells from human leukemia (Fig. 2J).

Taken together, we define the mode of action of Ramotac‐1, the first reported ATR degrader, and show that it promotes cytotoxic effects of DNA replication stress in cell lines and primary leukemic cells. Abd110 is based on VE‐821 and recruits CRBN via the lenalidomide part (Fig. 1A). The notion that Abd110 does not increase lethal effects of hydroxyurea in MOLT‐4^∆CRBN^ cells suggests that anti‐tumoral, DNA‐damaging effects of Abd110 rely on ATR degradation. This implies that Abd110 is a pure ATR degrader that lacks ATR kinase inhibitor activity. These data correspond to a weak binding of Abd110 to ATR [12] which is though sufficient to evoke its degradation in cells.

## 4 Conclusion

As prerequisite for life and the maintenance of homeostasis, DNA replication must be tightly controlled. The serine/threonine kinase ATR slows down the cell cycle and initiates DNA repair when DNA replication is disturbed, and genomic integrity becomes endangered. We reveal that the targeted pharmacological elimination of ATR provokes the still incompletely understood process of DNA replication catastrophe and that this eliminates leukemic cells. A PROTAC that specifically eliminates the pivotal cell fate regulator ATR allows new avenues of research that address how inhibition versus elimination of ATR affect cell fate upon DNA replication stress.

## Conflict of interest

OHK declares patents WO2019/034538, WO2016020369A1, WO/2004/027418, and advisory work for BASF Ludwigshafen, Germany. None of these patents covers substance classes in this work. BASF has not promoted, sponsored, or supported this study and its products are not discussed herein.

## Author contributions

AGK, RA, AMM, YZ carried out experiments. AA synthesized inhibitors. MPR contributed patient material. OHK, MB, WS designed the experiments. OHK wrote the paper.

### Peer review

The peer review history for this article is available at https://www.webofscience.com/api/gateway/wos/peer‐review/10.1002/1878‐0261.13638.

## Supporting information


**Fig. S1.** Analysis of the cell cycle phases of RS4‐11 cells after treatment with 1 μm Abd110 for 24, 48, 72 h.

## Data Availability

The source data underlying this article are available in the Supporting Information or will be provided by the corresponding author upon reasonable scientific request.
