# Supplementary material for: Pharmacological degradation of ATR induces antiproliferative DNA replication stress in leukemic cells
Source: Mol Oncol. 2024 Mar 22;18(8):1958–65. doi: 10.1002/1878-0261.13638 (PMC11306515; doi:10.1002/1878-0261.13638)

corresponding to fig 1b

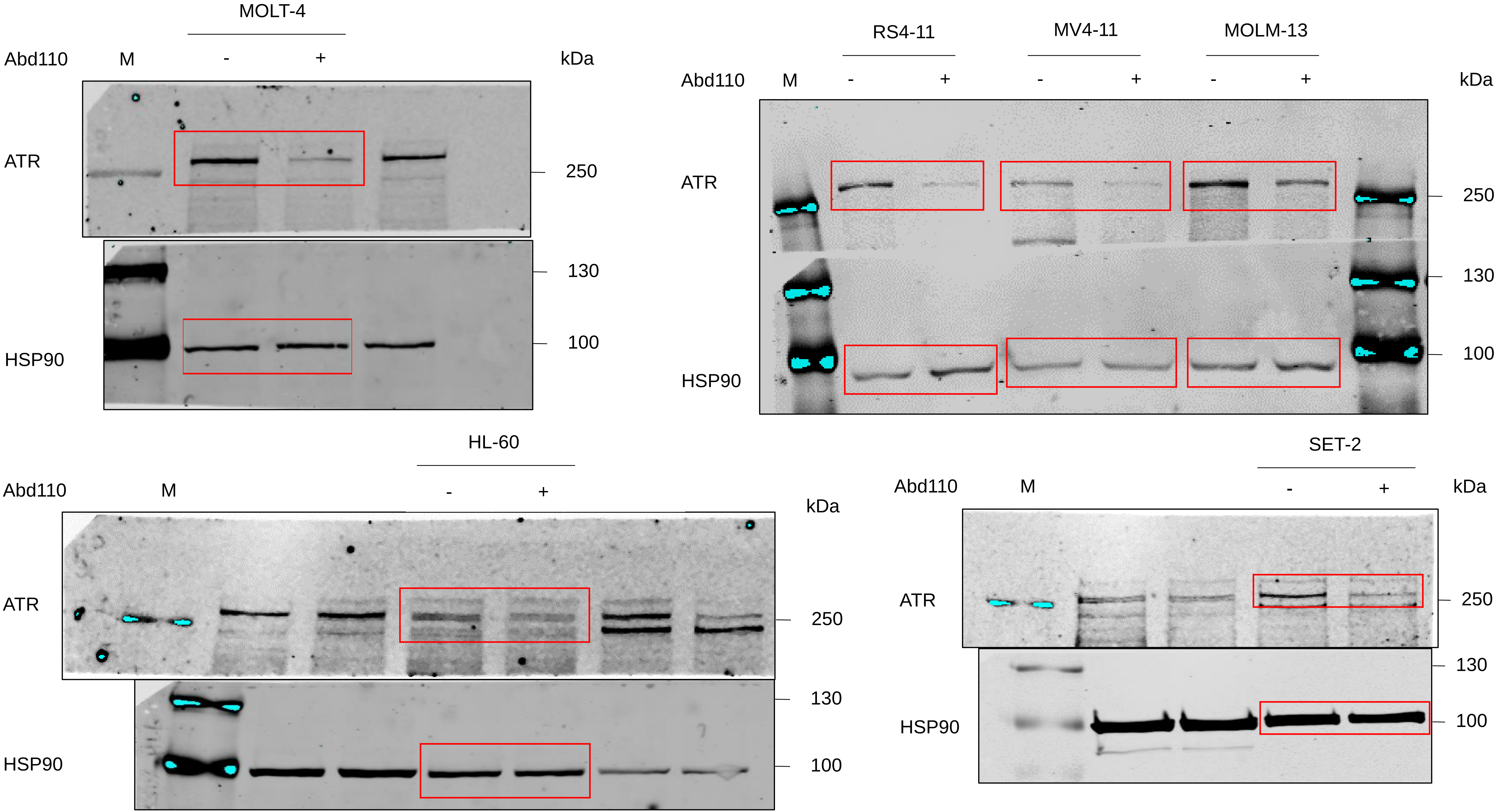

corresponding to fig 1c

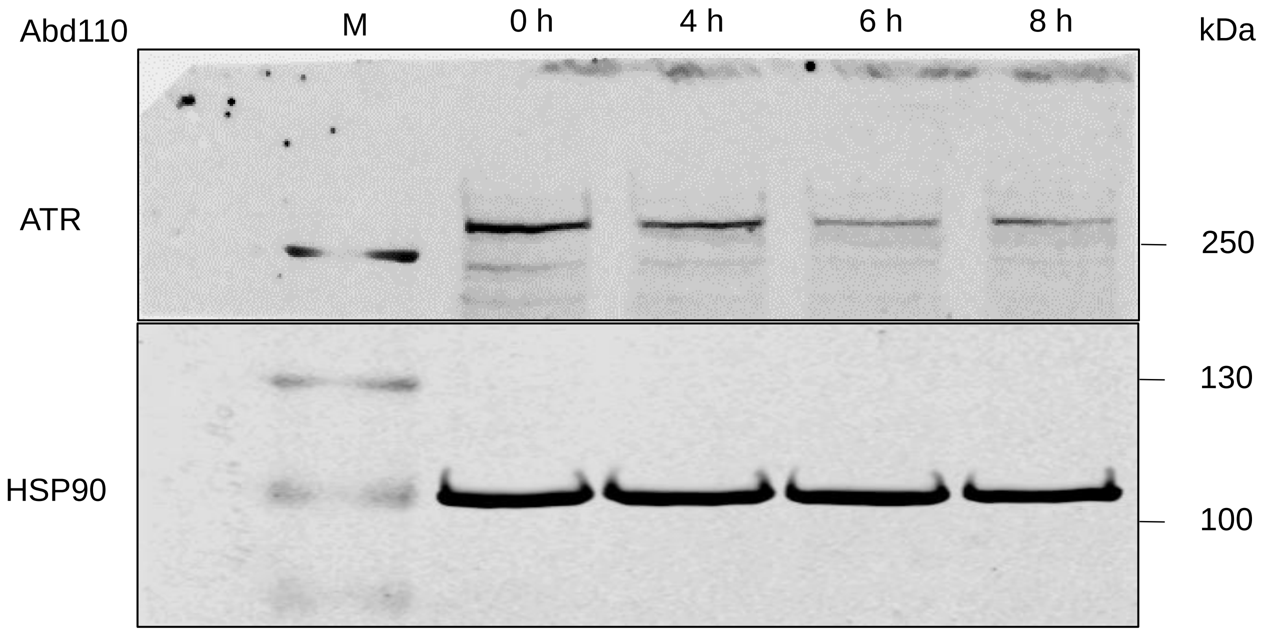

corresponding to fig 1d

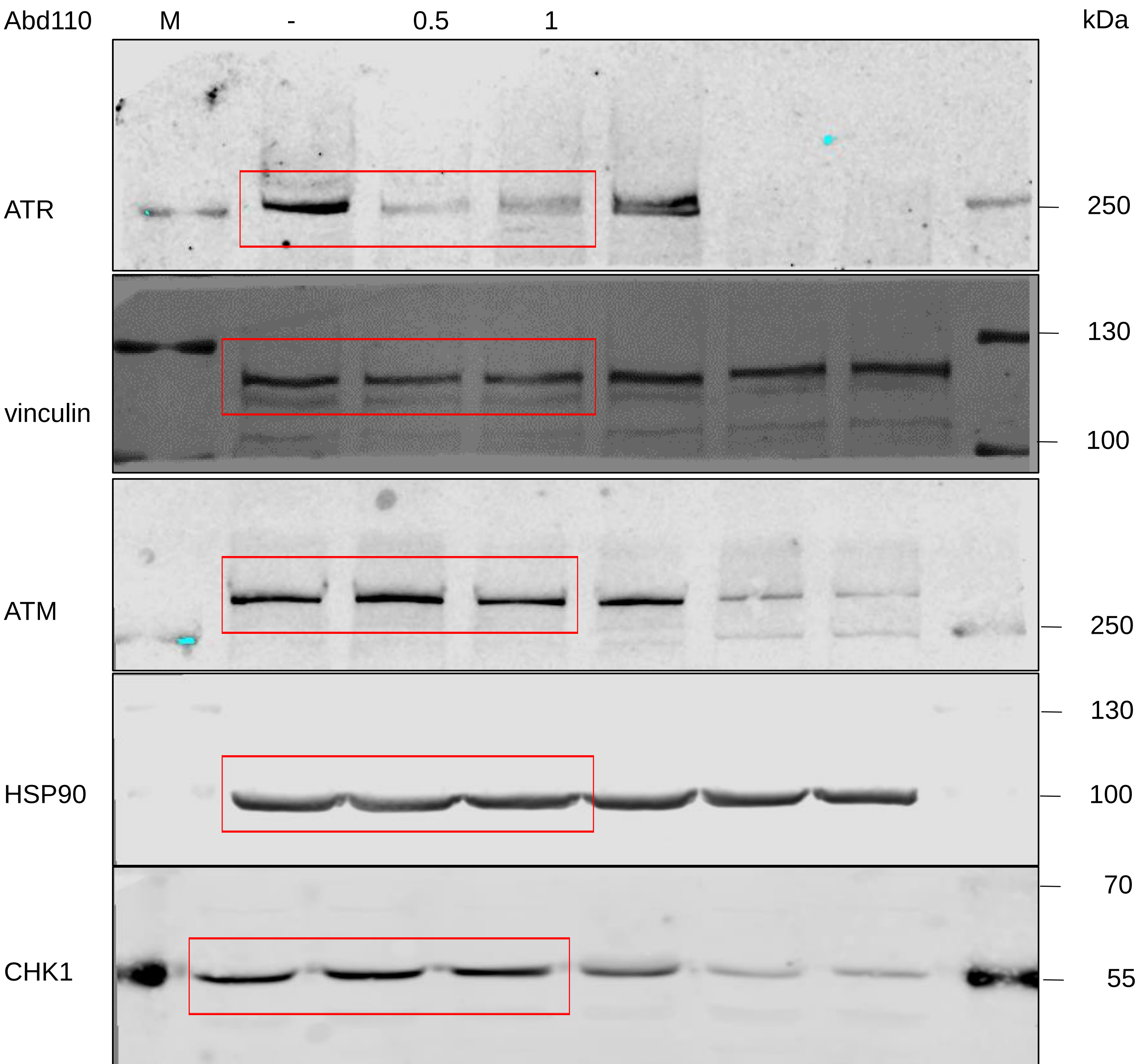

corresponding to fig 1e

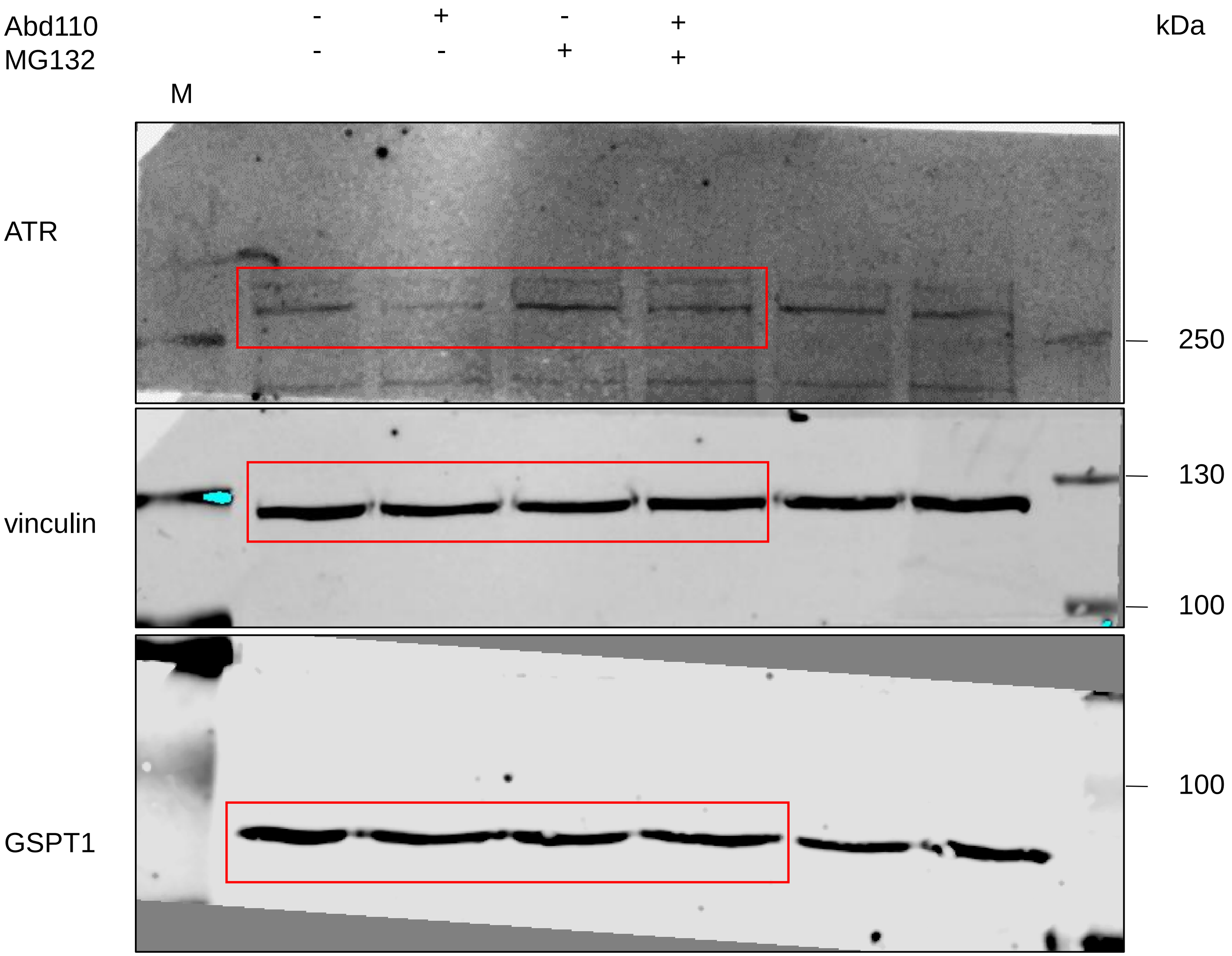

corresponding to fig 1g

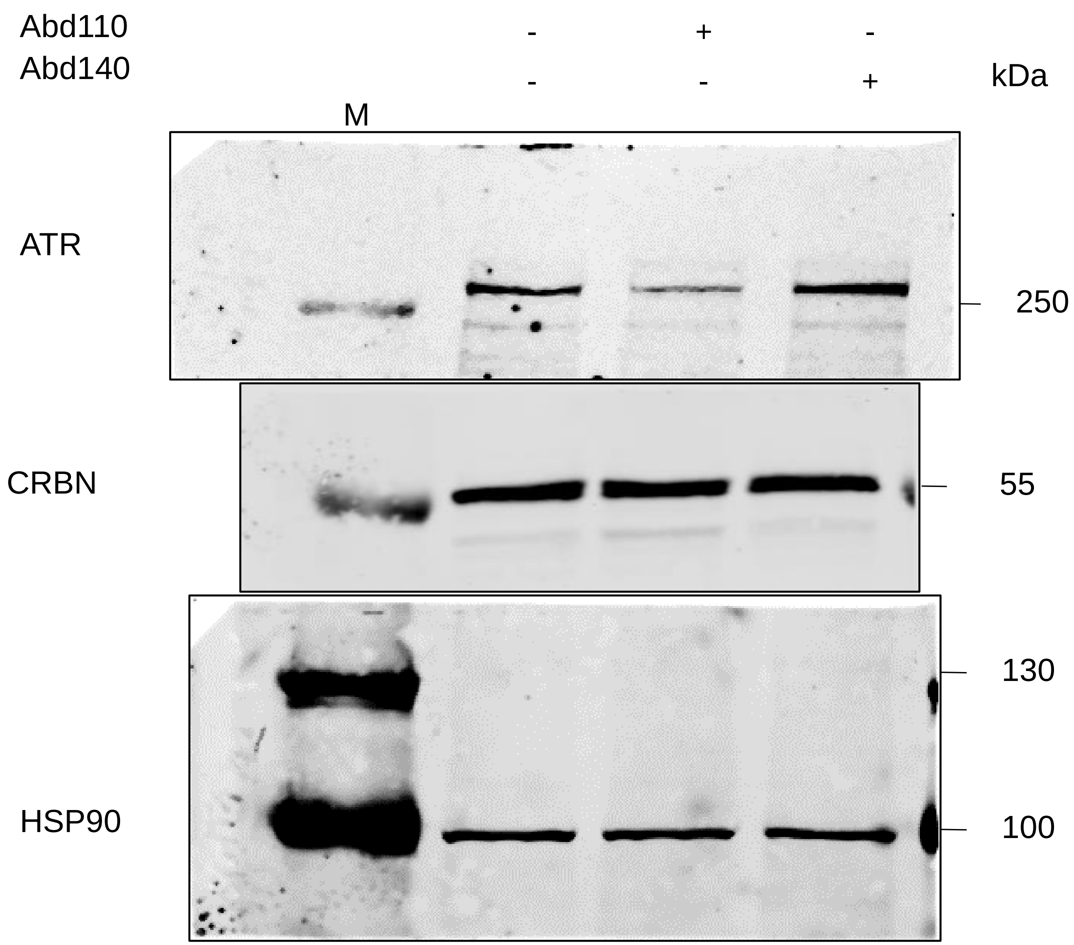

corresponding to fig 2a

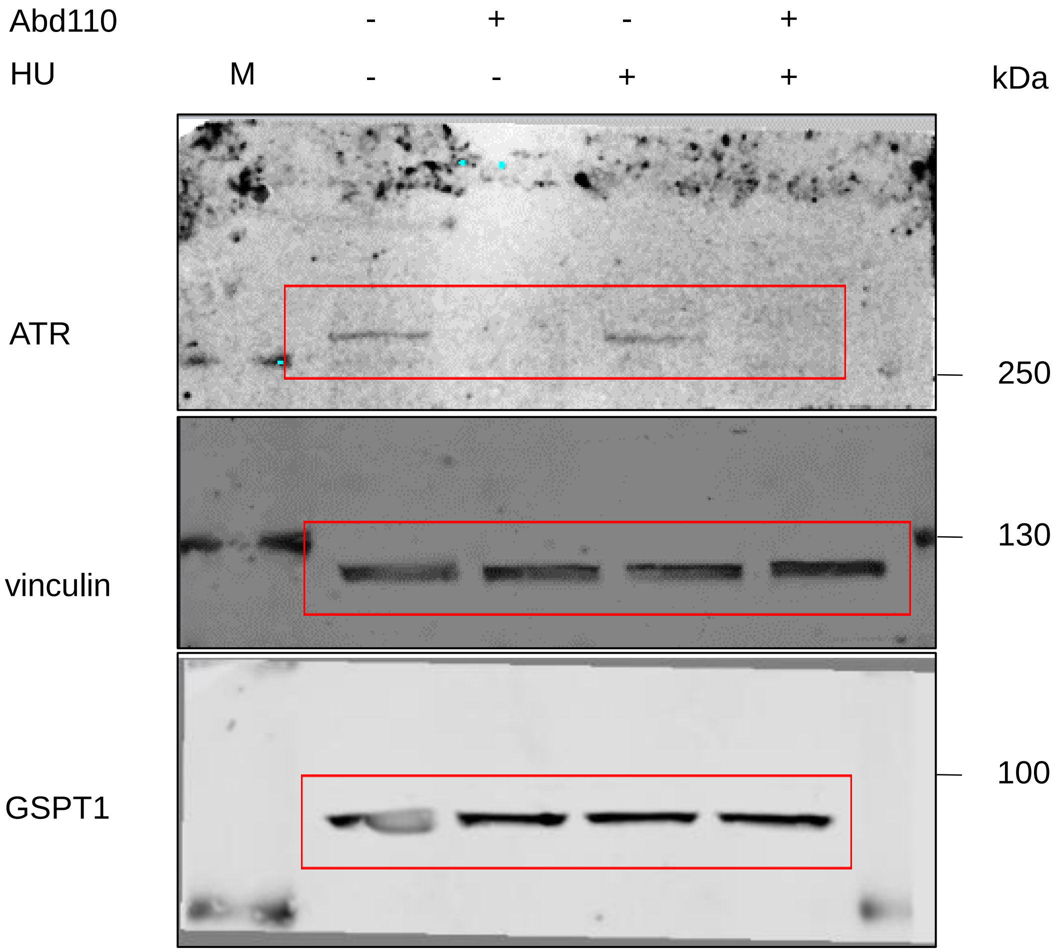

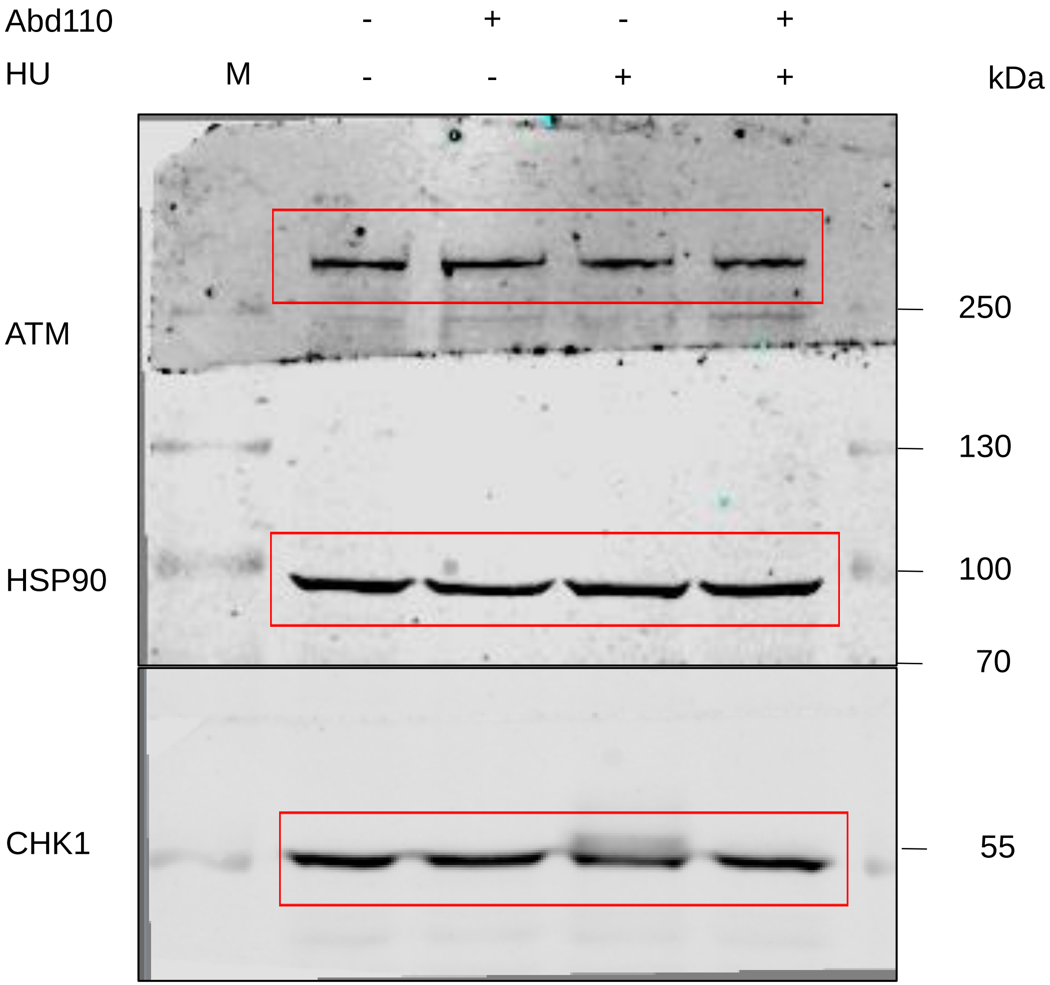

corresponding to fig 2a

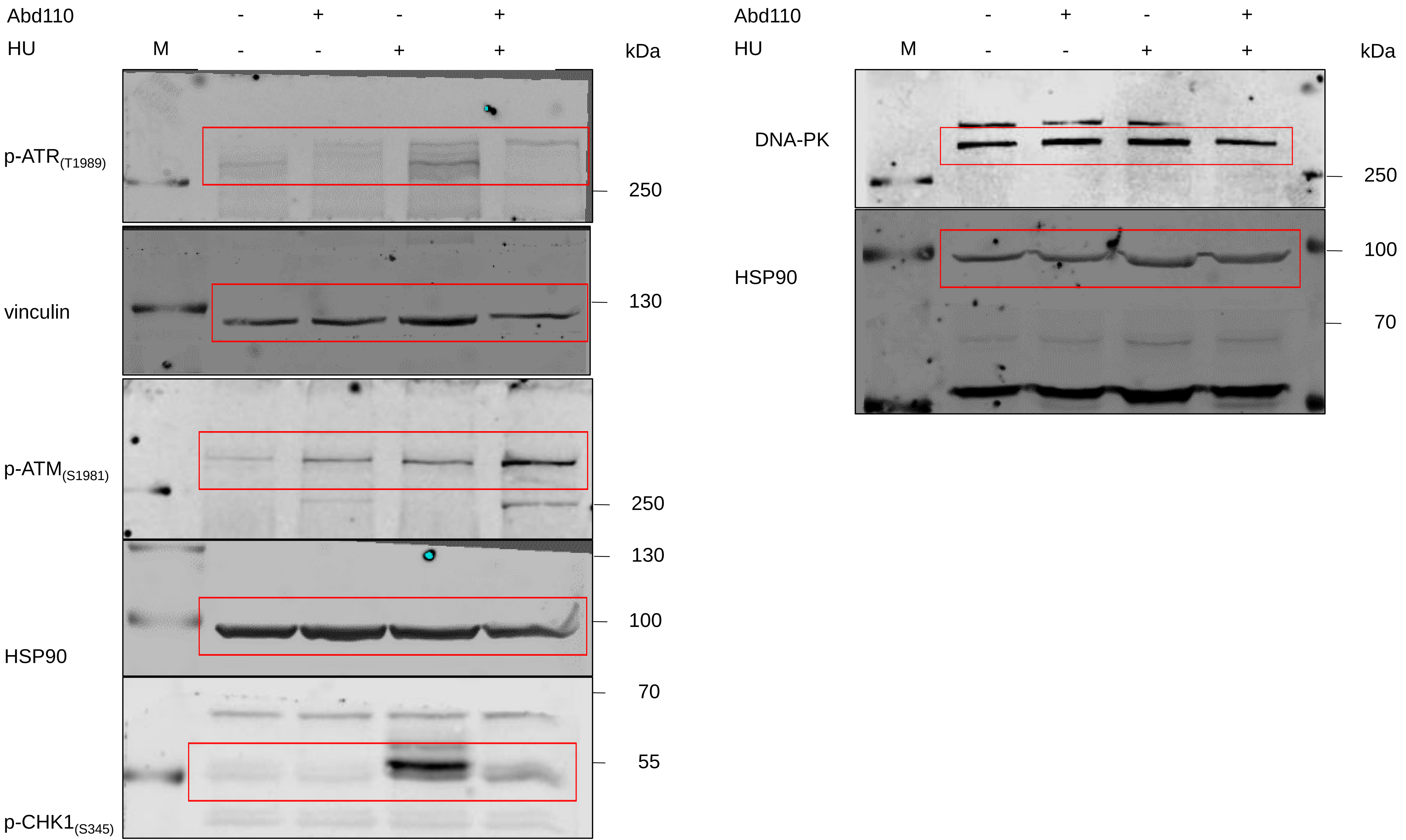

corresponding to fig 2c

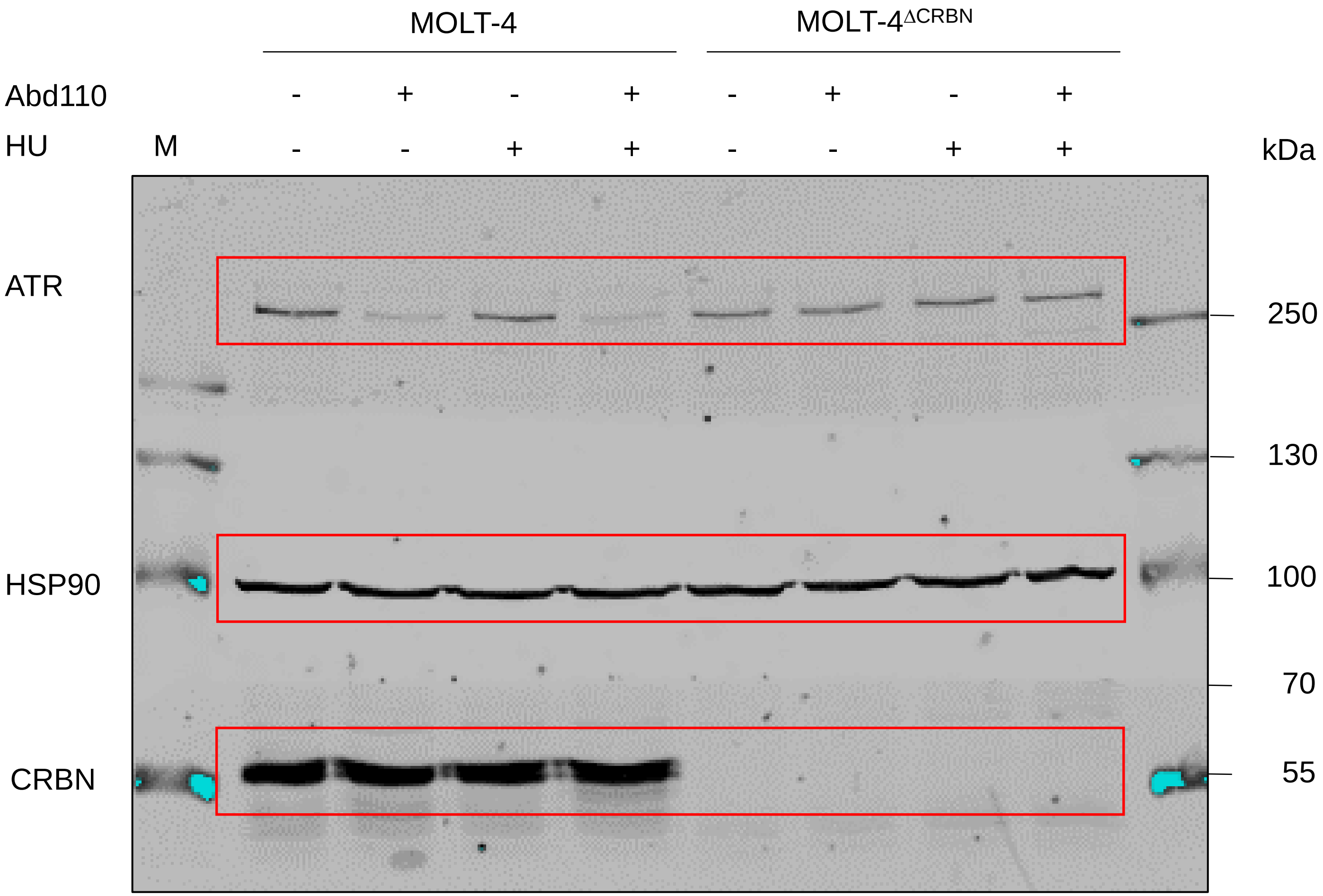

corresponding to fig 2c

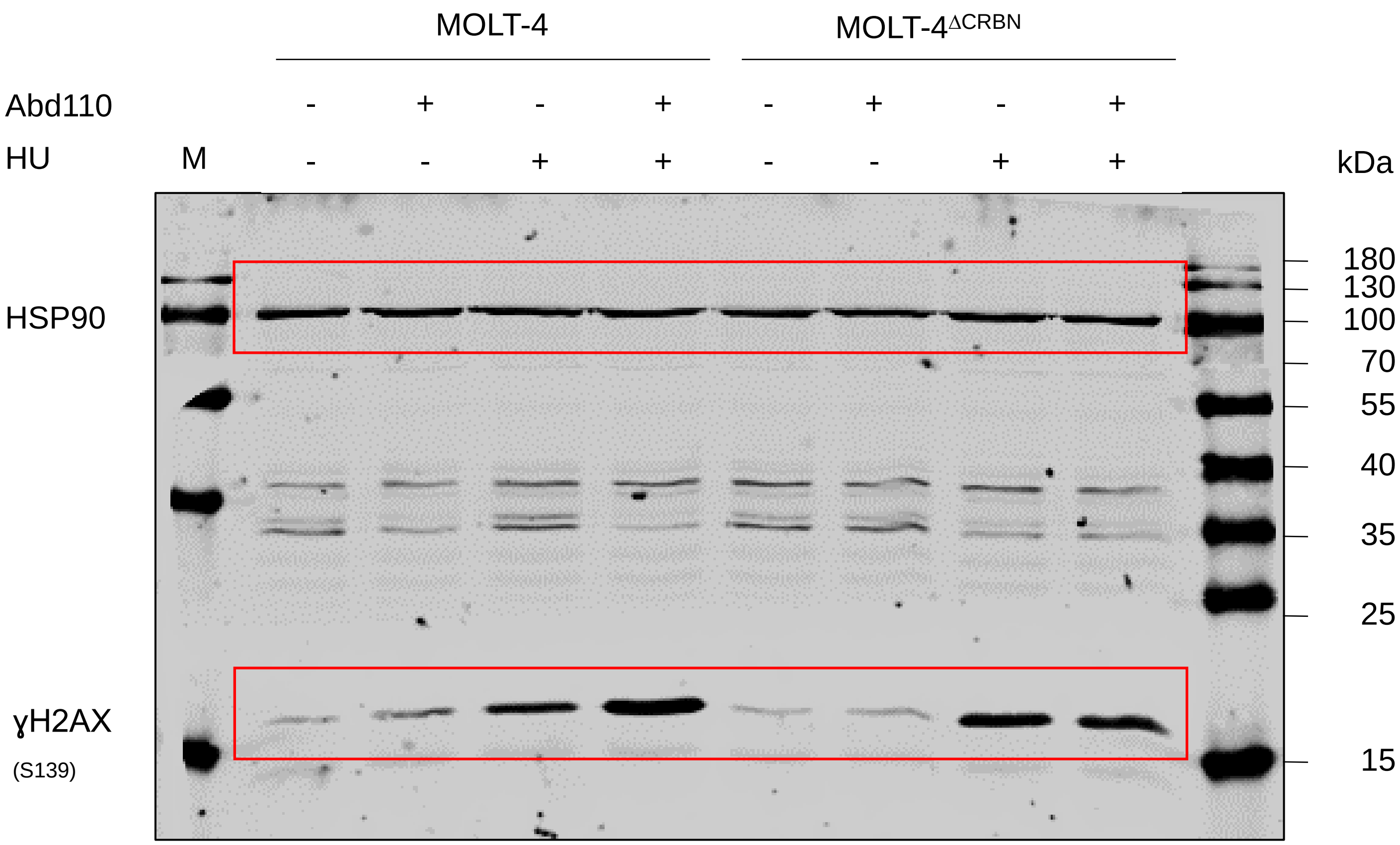

corresponding to fig 2e

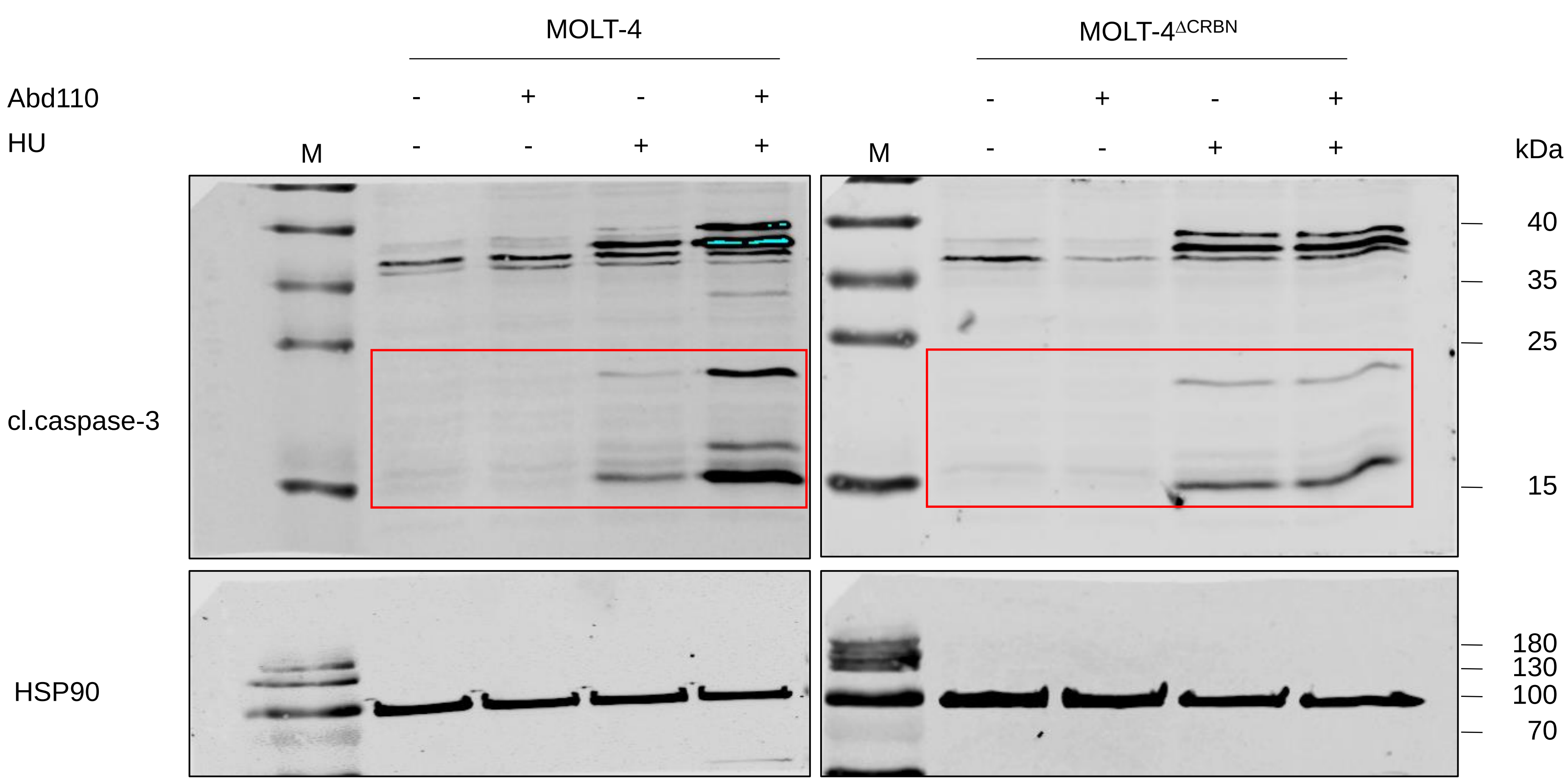

Supplement: Supplementary file 1 — Fig. S1. Analysis of the cell cycle phases of RS4‐11 cells after treatment with 1 μm Abd110 for 24, 48, 72 h. [file MOL2-18-1958-s001.zip › Original blots-Mol Oncol.pdf]
