# Supplementary figures and images for: Pharmacological degradation of ATR induces antiproliferative DNA replication stress in leukemic cells
Source: Mol Oncol. 2024 Mar 22;18(8):1958–65. doi: 10.1002/1878-0261.13638 (PMC11306515; doi:10.1002/1878-0261.13638)

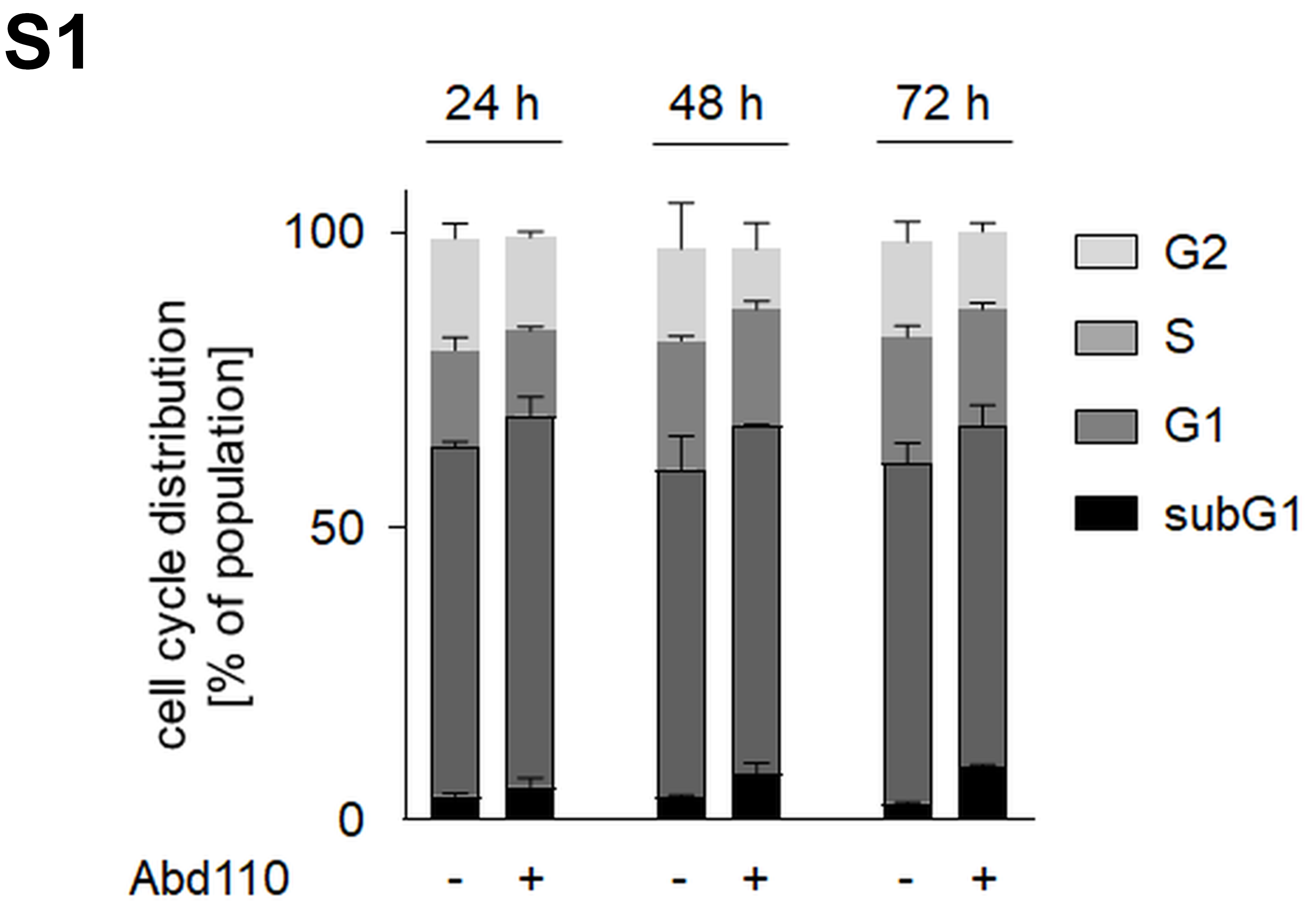

Supplement: Supplementary file 1 — Fig. S1. Analysis of the cell cycle phases of RS4‐11 cells after treatment with 1 μm Abd110 for 24, 48, 72 h. [file MOL2-18-1958-s001.zip › Figure S1.tif]
